# Development of *in planta* genome editing by transient expression of genome-editing tools in tomato

**DOI:** 10.3389/fgeed.2026.1777148

**Published:** 2026-04-09

**Authors:** Misaki Kobayashi, Na Renhu, Shu Takahashi, Seungje Choi, Haruto Watanabe, Martina Bianca Fuhrmann-Aoyagi, Hiroshi Ezura, Kenji Miura

**Affiliations:** 1 Graduate School of Life and Earth Sciences, University of Tsukuba, Tsukuba, Japan; 2 Tsukuba-Plant Innovation Research Center, University of Tsukuba, Tsukuba, Japan

**Keywords:** developmental regulators, genome editing, in planta, transient expression, Tsukuba system

## Abstract

Two major processes are important for genome editing in plants: transformation by stable transfection, in which nucleic acids encoding genome-editing enzymes are introduced into plant cells and the regeneration of plant individuals from cells harboring mutations by genome-editing enzymes. The efficiency of transformation and regeneration by tissue culture varies across plant species, and is low in some practical crop species. *In planta* methods have been developed to exclude the need for tissue culture. However, few reports are available on methods that do not require stable transfection. Therefore, this study aimed to develop a new protocol for delivery genome editing tools that does not require transformation or tissue culture, by combining the *in planta* method with transient genome editing tools instead of stable transfection. Cas9, guide RNAs, and developmental regulators, which are factors involved in mitotic tissue induction, were transiently expressed by agroinfiltration of the stem tissue cut surfaces of tomatoes. New chimeric mutants, containing a mixture of cells with mutations introduced at or near the target sequence, were obtained. After examining conditions such as the concentration of *Agrobacterium* used for infection and post-infection treatment, we succeeded in obtaining chimeric mutants with an efficiency of 11.7%. In addition, most of the observed mutations were single base substitutions. These results indicate that the *in planta* method with transient expression of genome editing tools and induction of meristematic tissue can be used to introduce genome-edited mutations in tomatoes.

## 1 Introduction

Genome editing uses artificial nucleases to induce double-stranded breaks in targeted DNA sequences, introducing mutations caused by repair errors in the broken gene. Genome editing employs site-directed nucleases to induce mutations in target genes with high precision and systematically alters traits. Breeding using genome editing can introduce mutations into target genes without changing the genotypes of elite varieties, thereby reducing the labor and time required for mutant selection and backcrossing. This approach makes it possible to develop new varieties in a few years compared to mutation breeding and backcross breeding, which take longer to reach commercialization (Ma et al., 2023).

Two important processes in plant genome editing technology are transformation by stable transfection, in which nucleic acids encoding genome-editing enzymes are introduced into the plant genome and tissue culture, in which individual plants are regenerated from cultured cells that have undergone mutation after transformation. Methods, such as *Agrobacterium* infection or particle bombardment techniques, which physically introduce DNA into cells, are commonly used (Zafar et al., 2020; Rahman et al., 2024)^,^. Successful genome editing of tomato, rice, maize, wheat, and soybean has been achieved using this two-step method (Abe et al., 2019; Zafar et al., 2020; Zheng et al., 2020; Aesaert et al., 2022; Choi et al., 2025). However, challenges are associated with both the processes.

Conventional genome-editing methods using *Agrobacterium* involve the introduction of vectors containing CRISPR-associated endonuclease 9 (Cas9) and guide RNA (gRNA) into plant cell nuclei, leading to the incorporation, or stable expression. This stable transfection allows foreign DNA to be maintained in the cell and its progeny over a long period. However, if genome editing tools remain in place for a long period of time, off-target effects will increase, and the organism will be subject to regulations on genetically modified organisms (Zhang et al., 2018). Therefore, after introducing a mutation, isolating null segregates in the progeny that harbor the mutation caused by genome editing, but do not contain the introduced foreign DNA, is necessary. In the *Agrobacterium* transformation method, VirD1 and VirD2 introduce a nick in the T-DNA on the Ti plasmid, releasing the T-DNA, and VirD2 attaches to the T-DNA ends, guiding it into the plant nucleus (Gelvin, 2017). Then foreign DNA is incorporated into the genome when T-DNA is integrated into the genome. Moreover, the efficiency with which they are incorporated into the genome varies between plant species and is difficult to achieve in species that are resistant to transformation, thereby limiting the number of species to which they can be adapted (Svitashev et al., 2016; Zhang et al., 2016; Fus-Kujawa et al., 2021).

Therefore, this study focused on transient rather than stable transfection. In transient transfection, transcription and translation begin as soon as foreign DNA reaches the plant nucleus; thus, foreign DNA is never incorporated into the genome (Voytas and Gao, 2014). In stable transfections, the number of DNA copies is low, leading to lower protein expression levels, whereas in transient transfection, a high copy number of the transfected genetic material may lead to high levels of protein expression (Fus-Kujawa et al., 2021). We previously developed base editing in tomato by transiently expressing nCas9, gRNA using the “Tsukuba system,” a high-level transient expression system and showed that transient expression of genome editing enzymes can allow mutagenesis (Yuan et al., 2021). The Tsukuba system contains a rolling-cycle replication system and a double terminator linked to heat shock protein and extension terminators. The expression of approximately 4 mg/g fresh weight of protein has been demonstrated in *Nicotiana benthamiana* using this system, achieving higher expression in 3 days (Yamamoto et al., 2018). Furthermore, the Tsukuba system, which harbors a geminivirus-derived replication system with a wide host range, allows expression in eggplant, tomato, pepper, lettuce, melon, orchid, soybean, bean, and radish, in addition to *N. benthamiana* (Yamamoto et al., 2018; Suzaki et al., 2019; Kitajima et al., 2020; Fuhrmann-Aoyagi et al., 2024).

Conventional genome editing using *Agrobacterium* is performed through tissue culture. However, the efficiency of individual regeneration through culture varies according to the plant species and is extremely low for practical crop species, making genome editing difficult for these crops (Hamada et al., 2018). Additionally, regeneration can take several months to complete. The *in planta* method was developed to solve varying regeneration efficiency and time-consuming processes in tissue culture by eliminating the need for tissue culture through direct transfection of plant tissues, such as flowers and growth points.

Research and development of stable transfection or tissue culture-free technologies are ongoing. *In planta* genome editing methods that do not require tissue culture have been developed using gRNA-expressing viruses or transient expression of developmental regulators (DRs); however, all of these approaches still require Cas9-expressing transgenic plants (Ellison et al., 2020; Maher et al., 2020; Li et al., 2021). As *Agrobacterium*-free methods, grafting and the sonication-assisted whisker method with RNP have been developed; however, the former requires Cas9-transgenic rootstocks, while the latter still requires tissue culture (Nakamura et al., 2023; Yang et al., 2023).

As described above, studies on stable transfection or tissue culture-free technologies have been conducted; however, few reports are available on methods that do not require either, for example *in planta* particle bombardment-RNP methods (Kuwabara et al., 2024). However, there are very few methods using the widely applicable *Agrobacterium* without stable transfection and tissue culture. Therefore, the aim of this study was to develop an *Agrobacterium*-mediated method that does not require stable transfection or tissue culture. To address this gap, we performed genome editing by combining a high-level transient expression system, the Tsukuba system, with an *in planta* method using meristematic tissue induction. The genome editing tools Cas9, gRNA, and DR were transiently expressed in tomatoes using *Agrobacterium*-mediated transient transfection technology without genomic integration. As a proof of concept, we targeted the autoinhibitory domain of *Solanum lycopersicum* glutamate decarboxylase 3 (*SlGAD3*), which led to a 15-fold increase in γ-aminobutyric acid content compared with the wild type (Nonaka et al., 2017). Chimeric mutants were obtained from the T_0_ generation. These results indicate that this strategy enabled the generation of genome-edited T_0_ mutant plants without stable transfection or tissue culture, demonstrating its potential as an effective alternative for genome editing in crops species were transformation and tissue culture are challenging. A detailed comparison of current genome-editing strategies is also discussed.

## 2 Materials and methods

### 2.1 Plant material

Tomatoes, *S. lycopersicum* cultivar Sicilian Rouge was obtained from Sanatech Life Science Co., Ltd, Tokyo, Japan. Seeds were sown in plastic pots with soil and grown at 24 °C, 16 h light/8 h dark. Seedlings approximately four to 7 weeks after sowing were used for infection.

### 2.2 Vector construction

**FIGURE 1:**
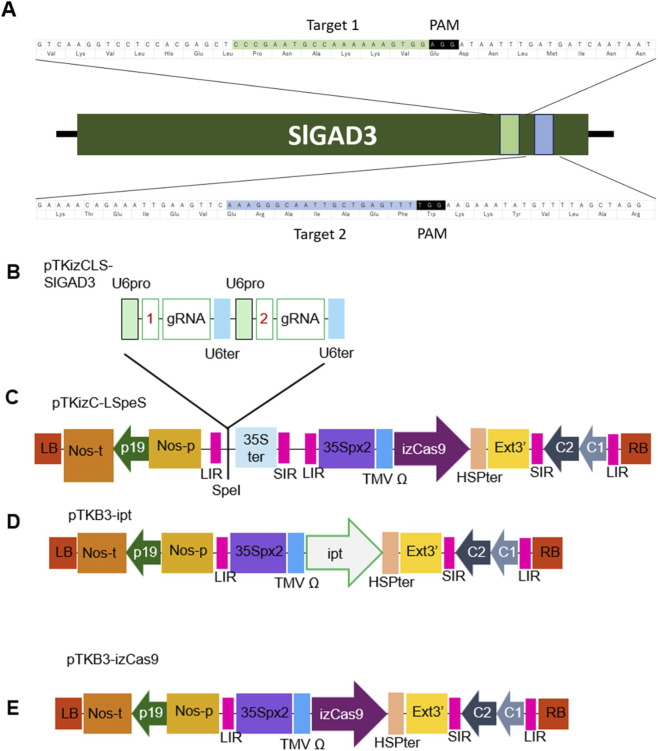
Schematic of the T-DNA region of plasmids **(A)** Target sites 1 and 2 of SlGAD3 PAM, protospacer-adjacent motif; NGG. **(B)** pTKizCLS-SlGAD3, **(C)** pTKizC-LSpeS, **(D)** pTKB3-ipt, and **(E)** pTKB3-izCas9 used for transient expression in tomato. LB and RB, the left and right border of the T-DNA region; Nos-p and Nos-t, NOS promoter and terminator; p19, a gene-silencing suppressor gene from tomato bushy stunt virus; LIR, long intergenic region of bean yellow dwarf virus (BeYDV) genome; SIR, short intergenic region of BeYDV genome; U6 pro, *Arabidopsis thaliana* U6-26 promoter; gRNA, guide RNA; U6 ter,U6 terminator; SpeI, restriction enzyme SpeI binding site; 35S-ter, CaMV 35S terminator; 35Sp×2, CaMV 35S promoter with double enhanced element; TMV Ω, 5′-leader sequence of tobacco mosaic virus; izCas9, Intronized *Zea mays*-codon usage Cas9; HSPter, heat stress protein gene terminator; Ext3′, tobacco extension gene 3′element; C1/C2, BeYDV ORFs C1 and C2 encoding for replication initiation protein, respectively.

Intronized *Zea mays*-codon usage optimized-Cas9 (izCas9) was synthesized using the gBlock Gene Fragment service (Integrated DNA Technologies), according to the sequence information (Grützner et al., 2021). When the DNA fragments were synthesized, the DNA sequences (5′- CTATTTACAAGTCGAACA -3′ or 5′- ATTCAGAATTGTCGAC -3′) were fused with izCas9 synthesized fragment at the 5′ or 3′ ends, respectively as an overlap sequence for In-Fusion cloning. The resulting DNA fragment was inserted into *Sal*I-digested pTKB3 (Nosaki et al., 2021) using the In-Fusion Snap Assembly Master Mix (Takara Bio). After confirmation of the insertion sequence, the vector was named pTKB3-izCas9 (Figure 1E).

The DNA fragment LIR-SpeI-35SterSIR, which contains a long intergenic region (LIR), *Spe*I restriction enzyme site, 35S cauliflower mosaic virus terminator (35Ster), and a short intergenic region (SIR), was also synthesized using the gBlock Gene Fragment Service. During synthesis, the DNA sequence 5′- GCGTGAAGCTGGCGCGCC -3′ was added to the 5′ end of the LIR and the DNA sequence 5′- TGCTAGAGCGGCGCG -3′ was added to the 3′ end of the SIR as an overlap sequence for In-Fusion cloning. The resulting DNA fragment was inserted into *Asc*I-digested pTKB3-izCas9 using In-Fusion Snap Assembly Master Mix. The resulting vector was named pTKizC-LSpeS (Figure 1C).

pTKC-LSGmPPD, which contains the U6-26 promoter (U6 pro), gRNA, and U6 terminator (U6 ter), was used as the template for gRNA cassette construction. First, three DNA fragments were amplified by PCR. The fragments corresponded to (1) U6 pro-SlGAD3 target1, (2) SlGAD3target1-gRNA-U6 ter-U6 pro-SlGAD3 target2, (3) SlGAD3 target2-gRNA-U6 ter. These three fragments were amplified using the primer pairs (1) pTKCL-U6-F and U6-26-SlGAD3-1-R, (2) SlGAD3 guideRNA F and U6-26-SlGAD3-2-R, (3) SlGAD3-2 guideRNA-F and pTKCL-U6end-R, respectively (Supplementary Table S1). The target sites were at 3′ region of *SlGAD3* to eliminate autoinhibitory domain (Figure 1A). Next, the three PCR fragments were used as templates to generate a single combined DNA fragment containing both gRNA cassettes by overlap PCR using outer primers pTKCL-U6-F and pTKCL-U6end-R. Finally, the resulting DNA fragment was inserted into *Spe*I-digested pTKizC-LSpeS, and the resulting plasmid was designated pTKizCLS-SlGAD3 (Figure 1B). pTKizCLS-SlGAD3 was designed to express izCas9 under the control of the CaMV 35S promoter with double enhanced element (35Sp×2) and two gRNAs targeting the 3′region of SlGAD3 under the control of the U6 promoter. The vector contains the essential components LIR, the replication initiation protein/replication initiation protein A and double terminator required for the Tsukuba system, enabling high-level expression of izCas9 and gRNA.

The synthesized DNA fragment containing the *isopentenyl transferase* (*ipt*) gene was inserted into *Sal*I-digested pTKB3 (Nosaki et al., 2021) and the resulting plasmid was named pTKB3-ipt (Figure 1D). pTKB3-ipt was designed, similarly to pTKizCLS-SlGAD3, to express the gene under the control of the 35Sp×2.

### 2.3 Transformation into *Agrobacterium*

The vectors produced above were transformed into *Agrobacterium tumefaciens* GV2260 together with pBBRacdSgabT (Nonaka et al., 2019). The vector combinations used were pTKizCLS-SlGAD3 + pBBRacdSgabT and pTKB3-ipt + pBBRacdSgadT. After transformation, the cells were selected on L-broth plates supplemented with 50 mg/L kanamycin and 30 mg/L gentamycin.

### 2.4 *Agrobacterium* preparations for transient expression in tomato

*A. tumefaciens* GV2260 harboring pTKizCLS-SlGAD3 + pBBRacdSgabT or pTKB3-ipt + pBBRacdSgadT, was pre-cultured in 5–10 mL of L-broth liquid medium containing 50 mg/L kanamycin, 30 mg/L gentamycin at 28 °C for one to two nights. The pre-culture was incubated in L-broth liquid medium containing 50 mg/L kanamycin, 30 mg/L gentamycin, 200 µM acetosyringone, and 20 µM 5-azacytidine at 28 °C in the dark with shaking until saturated stage. After centrifugation at 1930 *g* for 15 min at 4 °C, *Agrobacterium* was resuspended in the infiltration buffer (10 mM MgCl_2_, 10 mM MES [pH 5.6], 200 µM acetosyringone, 20 µM 5-azacytidine, 800 mg/L L-cysteine, and 0.05% silwet-77). *Agrobacterium* harboring pTKB3-ipt + pBBRacdSgabT and *Agrobacterium* harboring pTKizCLS-SlGAD3 + pBBRacdSgabT were separately adjusted to the desired optical density (OD_600_) using CO8000 Biowave (Biochrom), and then mixed for agroinfiltration. In all experiments, the OD_600_ of the pTKB3-ipt strain was kept at 0.2–0.4. The OD_600_ of the pTKizCLS-SlGAD3 strain was tested at 0.8, 1.0, 1.2, and 1.6 during optimization. After optimization, the OD_600_ was fixed at 1.2 for subsequent experiments.

### 2.5 Transient expression on tomato via *Agrobacterium* infection

Lateral shoots were removed from mature tomato seedlings, the main stem was cut at 2-3 lateral branches from the apical meristem, and the stem was infected by infiltrating the cut surface with a solution containing *Agrobacterium* using a 31G needle and 1 mL syringe with 10–20 inoculations (Supplementary Figure S1). In the post-infection treatment groups, a liquid fertilizer, Hyponex (nitrogen: phosphate: potassium = 6:10:5), was added to the tray immediately after infection. Heat stress treatment was also given to the plants by placing them under 37 °C for 3 h in darkness daily for 4 days, starting from the fourth day after infection. Subsequently, the plants were grown at 24 °C under 16-h light/8-h dark and any lateral shoots that emerged from non-infected surfaces or infected shoot surfaces within 10 days were removed. Because the number of shoots that emerged varied among individuals even after infection, multiple plants were infected together and treated as one group. The optimization of *Agrobacterium* harboring pTKizCLS-SlGAD3 concentration (OD_600_ = 0.8, 1.0, 1.2, and 1.6), as well as the post-infection treatments (liquid fertilizer addition and 37 °C heat stress) and the non-post-infection control infections, were each performed once. For biological replication, the optimized *Agrobacterium* concentration and the condition including post-infection treatment were each performed in three independent infection groups to confirm reproducibility.

### 2.6 Detection of mutation

Therefore, each shoot was treated as an independent biological event, and genomic DNA was extracted from the true leaves of shoots growing on the infected surface at 10 days post-infection. Shoots that developed only leaves or stems without a visible shoot apical meristem were excluded from the analysis, and only those with a clearly identifiable growth point were subjected to genomic analysis. The extraction procedure followed a previously described protocol (Yuan et al., 2021), with modifications in the centrifugation step at 18,700 x g for 5 min. PCR amplification was carried out using KOD FX Neo (TOYOBO) with the supernatant as the template. *SlGAD3* was amplified using the SlGAD3-multiF and SlGAD3-check2R primers (Supplementary Table S2). PCR products of the target genes were cloned as previously described (Yuan et al., 2021). If a positive clone was obtained, the insert was amplified using AmpliTaq Gold 360 Master Mix (Thermo Fisher Scientific). The PCR products were treated with Illustra ExoProStar (Cytiva) or ExoSAP-IT (Thermo Fisher Scientific), and sequence analysis was performed to confirm the mutation.

### 2.7 Analysis of T_0_ mutants to identify vector insertions

To determine whether foreign genes were inserted in the T_0_ mutants, PCR analysis was performed using KOD FX Neo (TOYOBO). Primers were designed to detect the presence of pTKizCLS-SlGAD3 and pTKB3-ipt vectors, which were used for transient expression. PCR products were amplified using genomic DNA extracted from shoot true leaves as templates and subjected to electrophoresis on 0.8% agarose gels. The primers used for PCR are shown in Supplementary Table S3, and their positions in the vector map are shown in Supplementary Figure S2.

## 3 Result

### 3.1 Effect of *Agrobacterium* concentration on genome editing efficiency in tomato

**FIGURE 2:**
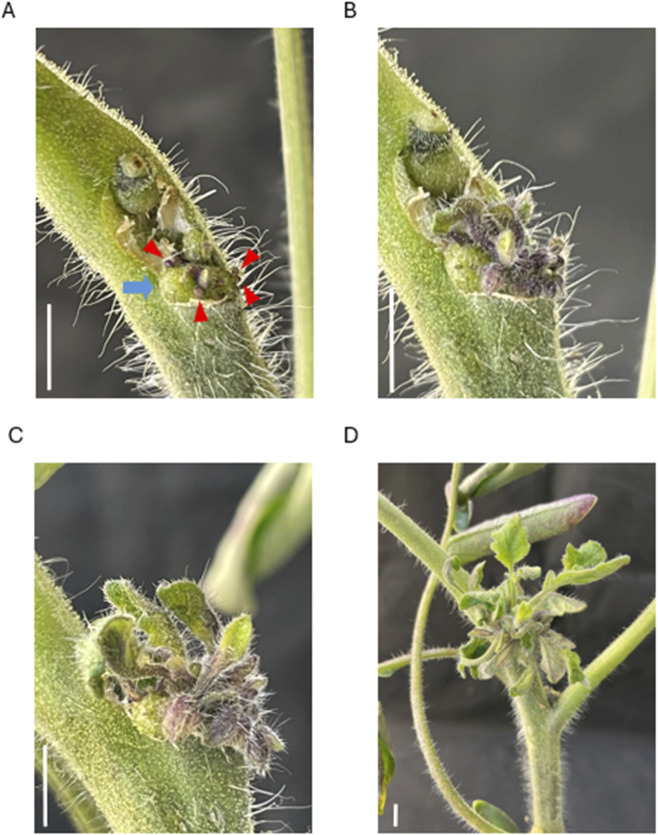
Shoots obtained by transient expression of izCas9, gRNA, and ipt in tomato Cross section of an *Agrobacterium*-infected stem. **(A)** 18 days after infection, shoot primordia emerged from the callus formed. **(B)** 20, **(C)** 22, and **(D)** 26 days later. Individual differences can be observed in the emergence and growth rate of the leaf stalks. Blue arrows represent callus, red triangles represent shoot primordia, and scale bars represent 5 mm.

**FIGURE 3:**
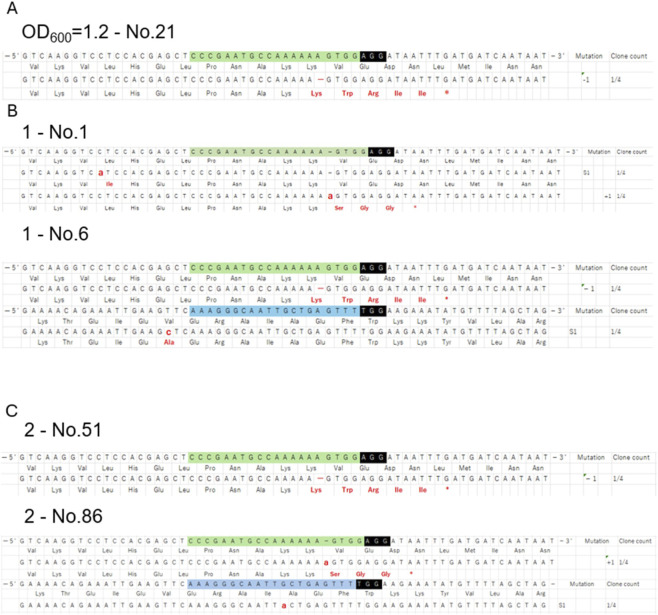
Sequencing results for mutants obtained in tomato. Sequencing results for the indel mutants from each experimental group are shown. Sequencing results for all mutants, including base substitutions, are shown in Supplementary Figure S3. **(A)** Experimental groups infected with *Agrobacterium* containing pTKizCLS-SlGAD3 at OD_600_ = 1.2; **(B)** post-infection treatment group (experiment1); and **(C)** sequencing results of indel mutants from biological replication experiment2 Green, target sequence (Target 1); blue, target sequence (Target 2); black box, PAM sequence; red, base and amino acid substitutions; mutation, type of mutation; S, base substitution; -, deletion; +, insertion; clone count, each individual four clones were cloned and used for sequencing analysis. The percentage of mutants among the analyzed clones is shown.

To introduce the mutation in *SlGAD3*, target1 and target2 of the *SlGAD3* gene (Figure 1A) were selected and the target sequence and gRNA were expressed under the U6 promoter. The *ipt* gene has been used to induce the differentiation of meristematic tissues through local overexpression as a type of DRs (Maher et al., 2020). When DR-free plasmids were used, neither callus nor shoot were obtained from wounded tomato stems (Lian et al., 2022). Since no shoots were generated when tomato stems were infected with genome-edited vectors of other target genes without *ipt* expression. For these reasons this study utilized the transient expression of *ipt* to induce meristematic tissue from the stem. The vectors pTKizCLS-SlGAD3 (Figure 1B) and pTKB3-ipt (Figure 1D) were transformed into *Agrobacterium* GV2260, together with pBBRacdSgabT, which had improved T-DNA transfer efficiency due to the addition of the ACC deaminase and GABA transaminase genes (Nonaka et al., 2019). These *Agrobacterium* solutions were mixed and infected with the cut surface of tomato stems to transiently express the genome-editing tools intronized and izCas9, gRNA, and ipt, a type of DR. Eighteen days after infection, purple shoot primordia emerged from the callus formed on the infected surface. In addition to shoots with apical meristematic tissue, leaf-only shoots arising from callus were also obtained (Figure 2). Shoots with apical meristematic tissue emerging 10 days after infection were designated as T_0_ lines and mutations caused by genome editing were detected. PCR fragments containing the target site were amplified, cloned into a cloning vector, and sequenced. Four clones from each T_0_ line were sequenced and if any of these clones had a mutation around target1 or target 2, they were categorized as genome editing mutants. To efficiently obtain genome-edited mutants using this procedure, the effect of *Agrobacterium* concentration on genome editing was investigated. Four concentrations of *Agrobacterium* harboring the vector pTKizCLS-SlGAD3, which transiently expresses genome-editing tools, were set at OD_600_ = 0.8, 1.0, 1.2, and 1.6 during infection. Several shoots were obtained from all four experimental groups, and sequence analysis revealed at least one mutation in all experimental groups (Table 1). The highest number of mutations was obtained when infected with an OD_600_ value of 1.2, with six lines, or 27.3% of the 22 lines classified as mutants. However, the probability of obtaining a mutation decreased to 17.6% (3/17) when the OD_600_ was 1.6. In addition, all mutation types detected in the mutants obtained from OD_600_ values of 0.8, 1.0, and 1.6 were single nucleotide substitutions, whereas at 1.2, a single nucleotide deletion was detected in one of the six mutant lines (Figure 3A; Supplementary Figure S3A). This mutation was speculated to cause a frameshift mutation, with the 451th amino acid being as the stop codon. The highest probability of obtaining a mutation among the four concentration conditions and the only insertion or deletion (indel) mutation detected suggested that the optimal concentration for *Agrobacterium* infection harboring pTKizCLS-SlGAD3 in this experiment was an OD_600_ value of 1.2.

**TABLE 1:** Summary of genome editing with different concentrations of *Agrobacterium* harboring pTKizCLS-SlGAD3.

Agrobacterium concentration OD_600_	No. of infected plants	No. of shoots	No. of T_0_ mutants (%)	Mutation type (chimeric rate) × No. of line	Target 1 (%)	Target 2 (%)
0.8	8	60	2 (3.3)	1 sub (1/4) ×2	2 (3.3)	0 (0)
1	8	32	1 (3.1)	1 sub (1/4) ×1	0 (0)	1 (3.1)
1.2	7	22	6 (27.3)	1 del (1/4) ×1 1 sub (1/4) ×5	3 (13.6)	3 (13.6)
1.6	7	17	3 (17.6)	1 sub (1/4) ×3	2 (11.8)	1 (5.9)

Mutants were defined as shoots obtained from the infected surface and wound in which at least one mutation was found among the four clones identified in the sequence. Mutation type indicates the type of mutation detected. The chimera rate indicated the number of mutations detected in the sequenced clones. The number of lines indicates the number of lines in which each mutation type was detected; target1 and target2 indicate the number of mutations detected in target1 and target2, respectively. Sequencing results for all mutants, including base substitutions, are shown in Supplementary Figure S3A.

### 3.2 Effect of post-infection treatments on the efficiency of genome editing in tomato

By transiently expressing genome-editing enzymes and ipt on the stem cut surface of tomatoes, chimeric genome-editing mutants were successfully obtained and the optimum concentration of *Agrobacterium* was determined. Therefore, we aimed to further improve the genome editing efficiency. Nitrogen is a key nutrient required for crop growth. In wheat, enzyme activity related to nitrogen metabolism, protein content, and yield were improved by applying fertilizer with optimized nitrogen levels (Wang et al., 2022). Therefore, we attempted to enhance post-infection plant growth and protein synthesis by applying nitrogen-containing liquid fertilizers after infection. In addition, since transient heat stress increases the cleavage activity of Cas9 and improves genome editing efficiency (LeBlanc et al., 2018), plants after *Agrobacterium* infection were placed at 37 °C for 3 h daily for 4 days from the day 4 post-infection. The number of shoots obtained per plant increased in the experimental group that was the addition of liquid fertilizer with heat stress, compared to those in the experimental group that was not treated after infection (non–post-infection treatment). The mutation acquisition efficiency was also slightly increased to 14.5% compared to that in 10.1% in the non–post-infection treatment group (Table 2). Furthermore, the mutants detected in the non–post-infection treatment group were single-nucleotide substitutions (Supplementary Figure S3B), whereas in the post-infection treatment group, two of the 11 mutant lines had indel mutations with the introduction of a stop codon due to a frameshift mutation (Figure 3B; Supplementary Figure S3B). To confirm reproducibility, two further experimental groups were prepared, in which the same conditions of liquid fertilizer and 37 °C heat stress were applied. Mutants were also obtained from two groups, one of which contained an indel mutation with the introduction of a stop codon (Table 3; Figure 3C). Three experimental groups with post-infection treatments were created, resulting in a total efficiency of 11.7% (30/257) of genome edits. Mutations detected in the three experimental groups were 7.0% (18/257) for target1 and 5.4% (14/257) for target 2. The mutation rate detected in target 1 tended to be slightly higher than target2, and indel mutations were detected only in target 1. Most mutant lines showed mutations in one of the target sequences, whereas some lines showed mutations in two target sequences simultaneously (Supplementary Figure S3C). The mutation types of the mutant strains obtained from the infected group with post-infection treatment also showed a high frequency of single nucleotide substitutions (Supplementary Figure S3B). Single nucleotide substitution mutations could have been caused by post-infection treatment itself or by errors in PCR or sequencing. Therefore, only *Agrobacterium* containing pTKB3-ipt was infected into tomato stems, performed post-infection treatment and the resulting shoots were analyzed. As a result of cloning sequencing four clones from each 37 shoots, a single base substitution suspected to be errors were found in only one shoot (Supplementary Figure S4). Based on the above, the probability of errors occurring was 2.7% (1/37), which is clearly lower than the mutation acquisition efficiency of 11.7% obtained in the reproducibility experiment of this study.

**TABLE 2:** Comparison of experimental and non–post-infection treatment groups with post-infection treatment.

Treatment	No. of infected plants	No. of shoots	No. of T_0_ mutants (%)	Mutation type (chimeric rate) × No. of line	Target 1 (%)	Target 2 (%)
Non–post-infection treatment	24	79	8 (10.1)	1 sub (1/4) ×8	3 (3.8)	6 (7.6)
Add liquid fertilizer and 37 °C stress (post-infection treatment)	13	76	11 (14.5)	1 in (1/4) ×1 1 del (1/4) ×1 1 sub (1/4) ×9	6 (7.9)	6 (7.9)

Mutants were defined as shoots obtained from the infected surface and wound in which at least one mutation was found among the four clones identified in the sequence. Mutation type indicates the type of mutation detected. The chimera rate indicated the number of mutations detected in the sequenced clones. The number of lines indicates the number of lines in which each mutation type was detected; target1 and target2 indicate the number of mutations detected in target1 and target2, respectively. Sequencing results for all mutants, including base substitutions, are shown in Supplementary Figure S3B.

**TABLE 3:** Summary of genome editing experimental groups subjected to post-infection liquid fertilizer and transient heat stress.

Experiment No.	No. of infected plants	No. of shoots	No. of T_0_ mutants (%)	Mutation type (chimeric rate) × No. of line	Target 1 (%)	Target 2 (%)
1	13	76	11 (14.5)	1 in (1/4) ×1 1 del (1/4) ×1 1 sub (1/4) ×9	6 (7.9)	6 (7.9)
2	13	92	8 (8.70)	1 del (1/4) ×1 1 in (1/4) ×1 1 sub (1/4) ×6	7 (7.6)	2 (2.2)
3	13	89	11 (12.4)	1sub (1/4) ×11	5 (5.6)	6 (6.7)
Total	39	257	30 (11.7)	​	18 (7.0)	14 (5.4)

Mutants were defined as shoots obtained from the infected surface and wound in which at least one mutation was found among the four clones identified in the sequence. Mutation type indicates the type of mutation detected. The chimera rate indicated the number of mutations detected in the sequenced clones. The number of lines indicates the number of lines in which each mutation type was detected; target1 and target2 indicate the number of mutations detected in target1 and target2, respectively. Sequencing results for all mutants, including base substitutions, are shown in Supplementary Figures S3B, S3C-Experiment 1.

### 3.3 T_0_ mutant shoots obtained via transient expression do not incorporate foreign genes

To confirm that no foreign genes were incorporated into the transiently expressed shoots, PCR analysis was performed to detect the presence of pTKizCLS-SlGAD3 and pTKB3-ipt vectors. Residual vector DNA was identified in the five indel mutants obtained in this study. No bands comparable to those in the positive control were detected in any of the five mutants (Supplementary Figures S2, S5). These results suggested that the transiently expressed foreign genes were not integrated into the genome in the five mutant shoots.

### 3.4 Mutation patterns were varied in the transient and *in planta* genome editing

Owing to tomato genome editing, multiple mutations have been detected in some mutant lines. Therefore, to determine more precise mutation types and chimera rates, an additional 16 clones were sequenced for the 11 mutant lines obtained from experiment 1 group of tomatoes. Although the number of clones sequenced to determine mutation patterns and chimerism varies among studies (Yu et al., 2017; Ma et al., 2019; Huang and Wang, 2021; Poovaiah et al., 2021; Yuan et al., 2021), we considered that analyzing 20 clones per line was sufficient in this study. Several additional mutations were detected in three lines, 1-1, 6, and 43, as shown in Table 4. The highest chimera rate was 25%. Mutation types varied, and the same mutation was not detected in all 20 clones sequenced (Supplementary Figure S6). These results suggest that various types of mutations are present in the T_0_ mutant; however, the chimera rate for each mutation type was as low as 5% (1/20).

**TABLE 4:** Summary of chimera rates of T_0_ mutants obtained in tomato.

T_0_ mutant	No. of mutant clone	Mutation type	Total chimeric rate
1-No.1	2	1in ×1 1sub ×1	10% (2/20)
1-No.3	1	1sub ×1	5% (1/20)
1-No.4	1	1sub ×1	5% (1/20)
1-No.5	1	1sub ×1	5% (1/20)
1-No.6	5	1 del×2 1 sub×3	25% (5/20)
1-No.10	1	1sub ×1	5% (1/20)
1-No.15	1	1sub ×1	5% (1/20)
1-No.20	1	1sub ×1	5% (1/20)
1-No.23	1	1sub ×1	5% (1/20)
1-No.29	1	1sub ×1	5% (1/20)
1-No.43	3	1 sub ×3	15% (3/20)

A total of 20 clones were sequenced in the 11 mutant lines obtained in experiment 1 with post-infection treatment. The number of mutant clones obtained from 20 clones, the type of mutation, and the chimera rate are indicated. The specific sequence results are shown in Supplementary Figure S6.

## 4 Discussion

In this study, the *in planta* method with transient expression of genome-editing enzymes and induction of meristematic tissue from stems introduced chimeric mutations via genome editing in the T_0_ generation of tomatoes.

Since the development of CRISPR/Cas9, many genome-editing methods have been reported, but few approaches have achieved both stable expression and tissue culture-free procedures. A comparison of current genome editing approaches was summarized in Table 5. There are several *in planta* methods that do not require tissue culture or integration of gRNA, but most still require Cas9 transgenic plants (Ellison et al., 2020; Maher et al., 2020; Li et al., 2021). This is likely because Cas9 is larger than gRNA or DRs, making transient expression more difficult. To develop a protocol that could potentially be applied to species that are difficult to transform, a system that does not require stable transfection of Cas9 was necessary. Therefore, in this study, we used the Tsukuba system, which is optimized for high-level transient expression, to also achieve transient expression of Cas9.

**TABLE 5:** Summary of comparison of current genome editing approach.

Method	Requires stable transfection or integrated genome	Requires tissue culture	Plant regeneration method	Genome-editing tools	Delivery method	Plant species	References
Particle bombardment-mediated CRISPR/Cas9 stable transformation	Yes (T-DNA integrated into genome)	Yes	Embryogenic callus–based regeneration	CRISPR-Cas9	Particle bombardment	Rice	Zafar et al. (2020)
In planta particle bombardment-RNP method	No (RNP)	No	Shoot and root induction from cultured embryogenic axes	CRISPR-Cas9	Particle bombardment	Soybean	Kuwabara et al. (2024)
conventional *Agrobacterium* genome-editing	Yes (T-DNA integrated into genome)	Yes	Callus induction and regeneration via tissue culture	CRISPR-Cas9	*Agrobacterium*	Mayze, wheat, soybean, tomato	Aesaert et al. (2022); Abe et al. (2019), Zheng et al. (2020), Choi et al. (2025)
Efficient genome-eiditing using geminiviral replicons	Yes (T-DNA integrated into genome)	Yes	Regenerarion via tissue culture using cotyledon	CRISPR-Cas9	*Agrobacterium*	Tomato	Dahan-Meir et al. (2018)
Gene editing using RNA viruses and mobile single guide RNAs	Yes (gRNA via virus but need Cas9 transgenic plant)	No	Seeds were harvested from viral vector–infected soil-plants	CRISPR-Cas9	*Agrobacterium*	*Nicotiana benthamiana*	Ellison et al. (2020)
Tissue cluture-free genome eiting with BSMV-sg system	Yes (gRNA via virus but need Cas9 transgenic plant)	No	Seeds were harvested from viral vector–infected soil-plants	CRISPR-Cas9	*Agrobacterium*	Wheat	Li et al. (2021)
Plant gene editing through *de novo* induction of meristems	Yes (Although gRNA and DRs are transiently expressed, Cas9 transgenic plants are required)	No	*De novo* shoot induction mediated by DRs	CRISPR-Cas9	*Agrobacterium*	*Nicotiana benthamiana*	Maher et al. (2020)
The sonication-assisted whisker method with RNP	No (RNP)	Yes	Callus induction and regeneration via tissue culture	CRISPR-Cas9	The whisker method	Rice	Nakamura et al. (2023)
grafting of wild-type shoots to transgenic donor rootstocks	Yes (A transgenic rootstock expressing genome-editing tools is required)	No	grafting	CRISPR-Cas9	Movement of genome-editing tools from the rootstock	*Arabidopsis thaliana*	Yang et al. (2023)
Base editing with highly expressed transient system “Tsukuba system”	No (nCas9, gRNA were transiently expression)	Yes	Callus induction and regeneration via tissue culture	nCas9	*Agrobacterium*	Tomato	Yuan et al. (2021)
This study	No (Cas9, gRNA, ipt were transiently expression)	No	*De novo* shoot induction mediated by DRs	CRISPR-Cas9	*Agrobacterium*	Tomato	​

The reported genome editing methods were categorized by approach, and whether each method requires stable transfection and tissue culture is indicated.

With the Tsukuba system, following *Agrobacterium* infection, the region between twoLIRs undergoes circularization in the plant cell nucleus. This results in the replication of a large amount of single-stranded DNA facilitated by the replication initiation protein/replication initiation protein A, a geminivirus-derived DNA replication enzyme. The Tsukuba system achieves higher expression levels by incorporating double terminators, which suppress the reduction in expression caused by RNA interference due to read-through (Yamamoto et al., 2018). Consequently, high amounts of izCas9, gRNAs, and *ipt* can be transiently expressed in tomato cells, facilitating the acquisition of mutant shoots.

In this study, two gRNAs were designed for a single target gene. Target 1 showed a higher editing efficiency than target 2, and indel mutations were detected only at target 1 (Supplementary Figure S3; Figure 3). Interestingly, indel mutations were found exclusively at target1, suggesting that differences in gRNA sequences influence on-target activity in DNA cleavage (Konstantakos et al., 2022), thereby affecting mutation efficiency and mutation type at different target sites. Among the mutations, single nucleotide substitutions were predominant. Single nucleotide substitution mutations could also arise form errors during PCR or sequencing. The frequency of substitutions detected when only ipt was expressed as a control was markedly lower than the frequency observed when genome-editing tools were expressed. This indicates that although some substitutions may result from technical error, the single-base substitutions detected in this study are more likely to represent genuine mutations induced by genome editing. Additionally, several studies have reported high frequencies of substitution mutations detected through CRISPR/Cas9-based genome editing in plants. In onion, deep sequencing analysis revealed a 54.84% frequency of base substitutions (Mainkar et al., 2023). Base substitutions were also observed at high frequencies in soybean protoplasts (Sun et al., 2015). In rice immature embryos, substitutions were observed at a frequency of 25%–45% (Macovei et al., 2018). In friable embryogenic callus of cassava, substitutions were observed more frequently than indel mutations (Odipio et al., 2017). In cotton protoplasts, most detected mutations were single-base substitutions (Chen et al., 2017). Furthermore, in melon protoplasts and transgenic plants, most detected mutations were substitution mutations (substitutions accounted for up to 91%, followed by insertions (7%) and deletions (2%)), and the probability of substitution was significantly higher than that of indel mutations (Hooghvorst et al., 2019). In addition, since Cas9-induced base substitutions have been reported to occur within ∼60 bp of the Cas9 cleavage site (Hwang et al., 2020), the single-base substitutions detected within 20 bp of the target sequence in this study are also considered to be izCas9-mediated genome editing events. Typically, Cas9-mediated genome editing induces a double-strand break 3-4 bases upstream of the PAM sequence, which is subsequently repaired through non-homologous end-joining. Errors in this process often result in small indel mutations (Chauhan et al., 2023). The izCas9 variant used in this study contains Cas9 with13 additional introns. With izCas9, it is possible to generate large deletion mutations exceeding 1000 bases (Grützner et al., 2021). Nevertheless, several factors may explain the high frequency of single nucleotide substitutions observed in this study. The transient expression of Cas9 in this study may have led to the production of structurally incomplete Cas9 proteins, increasing the likelihood of nick formation due to incomplete DNA cleavage. Nicks are a form of DNA damage and play a role in various repair pathways, serving as intermediates in base excision repair, nucleotide excision repair, and mismatch repair pathways (Maizels and Davis, 2018). When nicks occur and corrective errors occur during these repair pathways, single nucleotide substitutions can arise (Krawczak et al., 1998). If transient nicks occurred in large numbers over a short period, the resulting repair mechanisms may have introduced corrective errors, leading to single nucleotide substitutions around the target region.

OD_600_ = 1.2 was the optimum concentration for the transient expression of genome editing tools in tomatoes in this study (Table 1; Supplementary Figure S3A). Even at a higher concentration, OD_600_ = 1.6, the mutant efficiency was higher than at 0.8 or 1.0, however, decreased compared to 1.2. A similar phenomenon as reported in a study of transient transformation in peony, where the efficiency of transient expression of the β-glucuronidase gene increased with increasing concentrations of OD_600_ = 0.8, 1.0 and 1.2, however, decreased at 1.4 (Guan et al., 2022). Furthermore, as the OD of Cas9-expressing *Agrobacterium* increased, the number of shoots tended to decrease (Table 1). The relative proportion of *Agrobacterium* expressing *ipt* may decrease when a higher OD of Cas9-expressing *Agrobacterium* is used. However, because the total OD of the infection solution was maintained within the range of 0.2–0.4, there was no substantial reduction in *Agrobacterium* carrying *ipt*. At the highest OD tested (1.6 for Cas9-expressing *Agrobacterium*), many plants exhibited withered and hardened infection sites. If the reduction in shoot induction were solely due to insufficient *ipt*, one would expect shoots to remain alive but fail to elongate. Instead, we observed tissue necrosis, suggesting that the decreased shoot induction rate was not caused by a shortage of *ipt*, but rather by the excessively high total bacterial concentration, which led to cell death at the infection sites. Based on the above, at low bacterial concentrations, infection efficiency is low, and a concentration that is too high will weaken the plant; thus, finding the optimum concentration for the plant is important.

In tomatoes, the addition of liquid fertilizer and transient 37 °C stress after *Agrobacterium* infection tended to increase the number of regenerated shoots, efficiency of mutation acquisition, and indel mutants obtained (Tables 2 and 3). The addition of liquid fertilizer may have promoted growth and increased the number of regenerated shoots. In addition, plants synthesize amino acids by absorbing nitrogen sources, such as ammonium and nitrate. Additional nitrogen sources are assumed to be required for transient expression. Since feeding nitrogen-containing fertilizer enhances nitrogen metabolism-related enzymes (Wang et al., 2022), the addition of liquid fertilizer may have increased the efficiency of transient expression of Cas9 and gRNAs, which in turn increased the number of indel mutants. However, additional experiments are needed to investigate this causal relationship.

Transient heat stress improves Cas9 cleavage activity (LeBlanc et al., 2018). In this study, applying heat stress also tended to increase the number of indel mutants. High yields of green fluorescent protein were obtained on day 3 post-infection in *N. benthamiana* (Yamamoto et al., 2018). In this study, heat stress was applied by placing the plants at 37 °C for 3 h daily for 4 days from the fourth day of infection. Applying heat stress continuously on days 3–7 of infection would be ideal, when transient expression peaks. However, heat stress has diverse effects on plants. In tomato, high temperature stress negatively impacts growth (Hoshikawa et al., 2021). When plants were exposed to 37 °C for 24 consecutive hours on the fourth day after infection, several individuals exhibited complete wilting. Similarly, previous studies have reported accelerated disease transmission in barley following several days of heat stress at 35 °C (Schwarczinger et al., 2021). In summary, prolonged heat stress can have various detrimental effects on plants. Therefore, the peak transient expression period of Cas9 in each plant species must be investigated and the period for intensive 37 °C treatment must be targeted.

As plant growth regulators, DRs are involved in the differentiation and maintenance of shoot apical meristem tissues through their interaction with cytokinins and auxins (Barton, 2010). DRs have been used to enhance plant regeneration and transformation efficiency in tissue culture and other plant-based methods (Maher et al., 2020; Lian et al., 2022). *ipt* acts in the early stages of cytokinin biosynthesis in plants and is used as one of the DRs to induce shoot organ formation through ectopic overexpression of *ipt* (Smigocki and Owens, 1988; Sakakibara et al., 2005; Maher et al., 2020). In this study, *Agrobacterium*-mediated expression of *ipt* as a DR in wounded tomato stems resulted in callus formation and shoot emergence. Similar results were obtained when *Arabidopsis thaliana* PLETHORA was expressed in wounded tomato stems, where shoots formed from the callus, despite different DRs being used (Lian et al., 2022). As this study used transient genome-editing enzyme expression, the analysis was performed without selection using drugs or other chemicals. As a result, mutants were obtained with an efficiency of up to 27%; however, many shoots without any mutations grew. Cytokinins synthesized by the *ipt* gene promote regeneration not only in the synthesized cells but also in the surrounding cells (Umemoto et al., 2023). This might explain the low chimera rates observed for each mutation type. Other DRs were not tested in this study. However, at least in this study where ipt was transiently expressed as DRs seems to be suitable for the genome editing of genes that show a visible phenotype when mutated. Several other genes besides ipt have been used as DRs, and their selection is very important. For example, the overexpression of *A. thaliana* PLETHORA or *A. thaliana* WUSHE at the stem injury site in tomatoes promotes shoot regeneration and transformation efficiency. However, when *A. thaliana* WOUND INDUCED DEDIFFERENTIATION 1 was used to produce transformed shoots in *Antirrhinum majus*, no transformed shoots were obtained in tomatoes (Lian et al., 2022). Thus, different plant species have different DRs that affect the transformation efficiency. Therefore selecting appropriate DRs for each plant species that not only promote shoot regeneration, but also improve transformation and genome editing efficiency, is important. When transient expression of gRNA and DRs was performed in *N. benthamiana* expressing Cas9, shoots showing reporter gene activity were obtained not only when *ipt* was expressed solely, but also when *Z. mays* WUSCHEL2 and *ipt* were co-expressed, and genome-edited shoots were obtained from those co-expressed (Maher et al., 2020). This suggests that the protocol used in this study can further improve genome editing efficiency by selecting appropriate DRs or coexpressing appropriate DRs in combination, depending on the plant species.

In this study, genome-editing mutations were successfully introduced into the T_0_ generation in tomato by transient expression of genome-editing enzymes and the *in planta* method using meristematic tissue induction. Normally, in the transformation and genome editing of tomatoes using tissue culture, more than 6 months are required to form flower buds after regeneration. In this system, flower buds are formed from regenerated shoots as early as 6 weeks after *Agrobacterium* infection of plants, saving 4 months compared to conventional methods. In addition, since the indel chimera mutant obtained by transient expression did not show persistence of the foreign gene (Supplementary Figures S2, S5), transgene-free next-generation individuals are expected to be obtained. The system in this study uses a highly versatile *Agrobacterium*-mediated method, and furthermore the Tsukuba system used in this study can be expressed in various plant species other than tomatoes (Yamamoto et al., 2018). Therefore, this system could be adapted to plant species for which the Tsukuba system can be adapted. Multiple variant types were detected from a single shoot, and the detailed chimera rate was 5%–25% (Supplementary Figure S6). Because the chimera rate for each mutation type observed in experimental group 1 was approximately 5%, we infer that a similar mutation frequency is likely to apply to other strains as well. Further improvements to the protocol are needed to increase the chimera rate and indel mutation rate. Further investigation and optimization of other factors affecting editing efficiency may make this protocol a highly efficient and versatile tool for genome editing.

## Data Availability

The original contributions presented in the study are included in the article/Supplementary Material, further inquiries can be directed to the corresponding author.
