# Supplementary material for: Development of in planta genome editing by transient expression of genome-editing tools in tomato
Source: Front Genome Ed. 2026 Apr 9;8:1777148. doi: 10.3389/fgeed.2026.1777148 (PMC13102611; doi:10.3389/fgeed.2026.1777148)
Supplement: Supplementary file 1 [file Supplementaryfile1.pdf]

Supplementary data

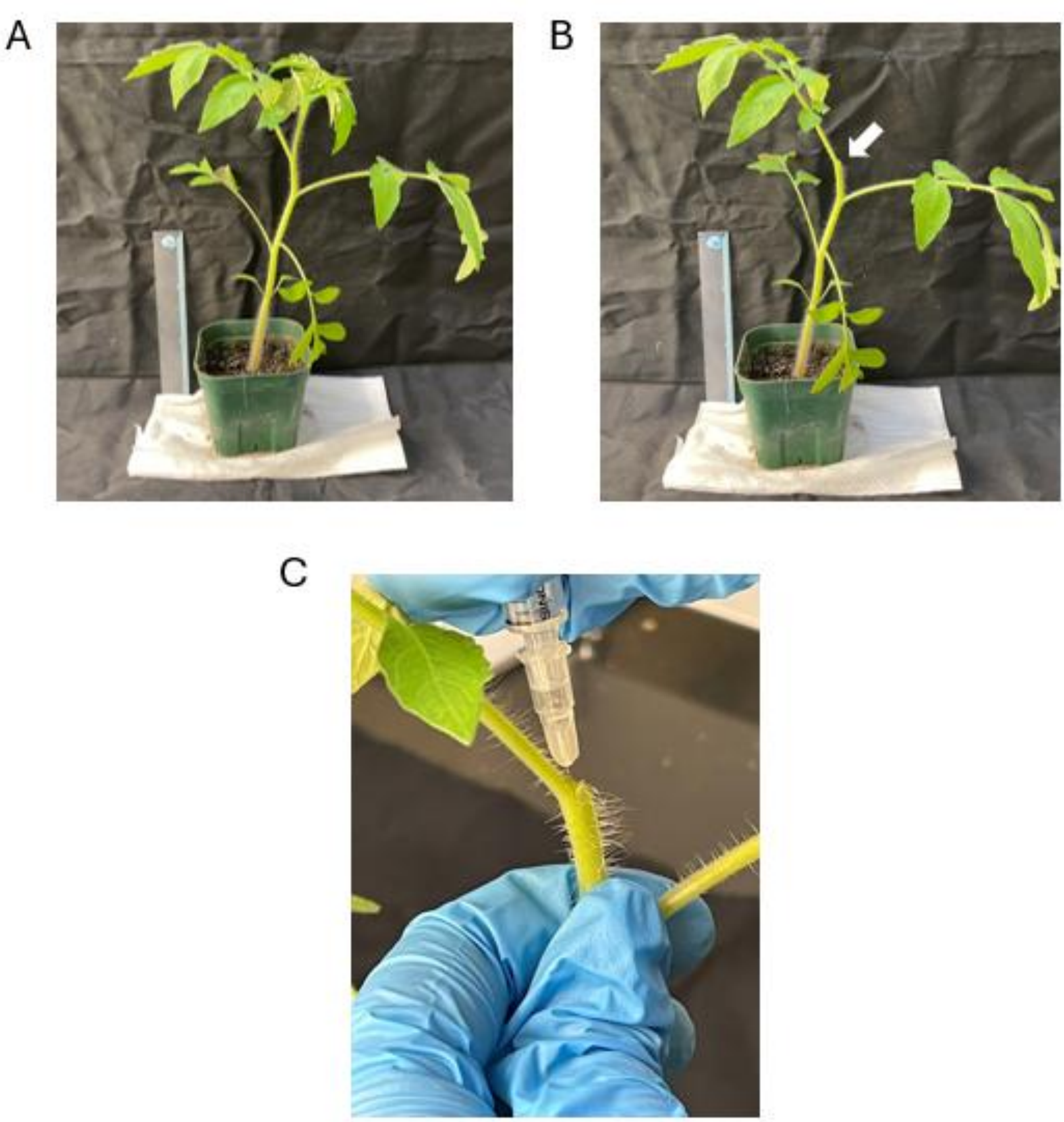

Supplementary Figure S1. Infection of tomato stems. (A) Side shoots were removed from seedling. The photo shows a seedling four weeks after sowing. (B) The main stem was cut at 2-3 lateral branches below the apical meristem. The white arrows indicate the severed part. (C) Agrobacterium was applied to the wound using a syringe while making 10-20 wounds with a 31G needle at the cut stem.

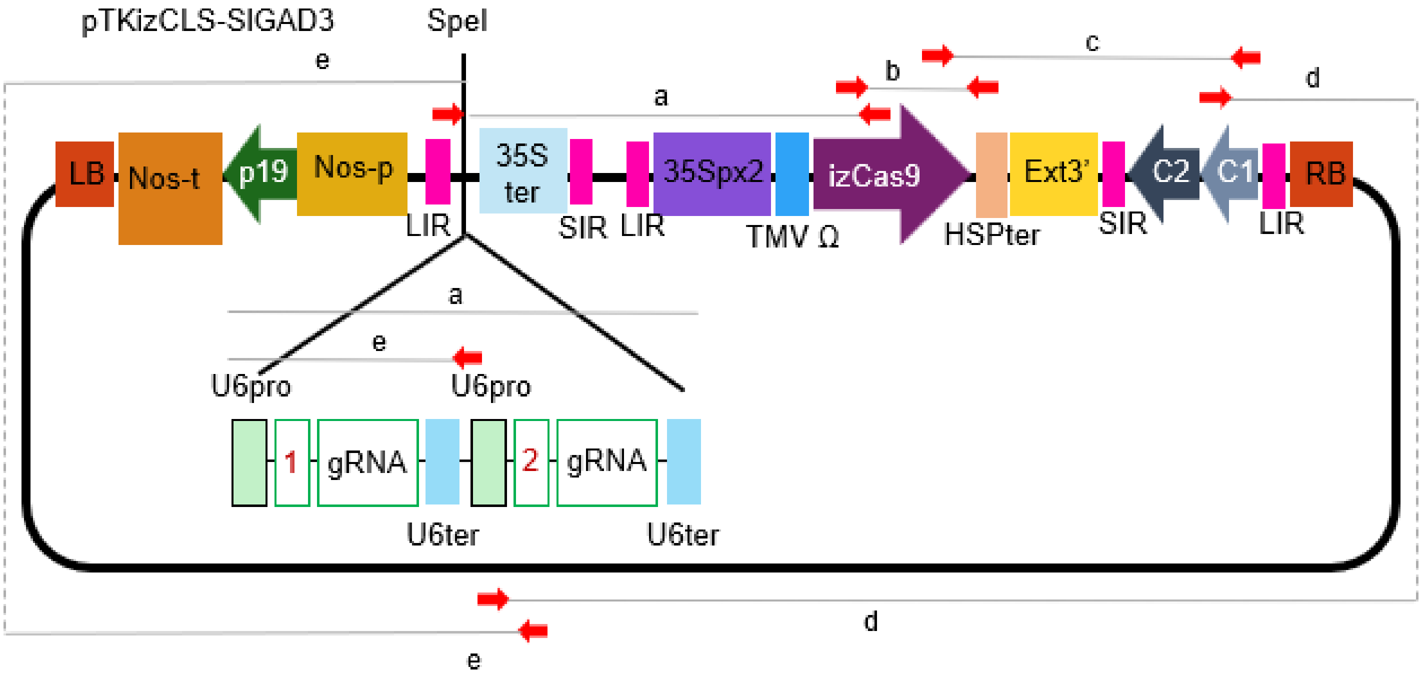

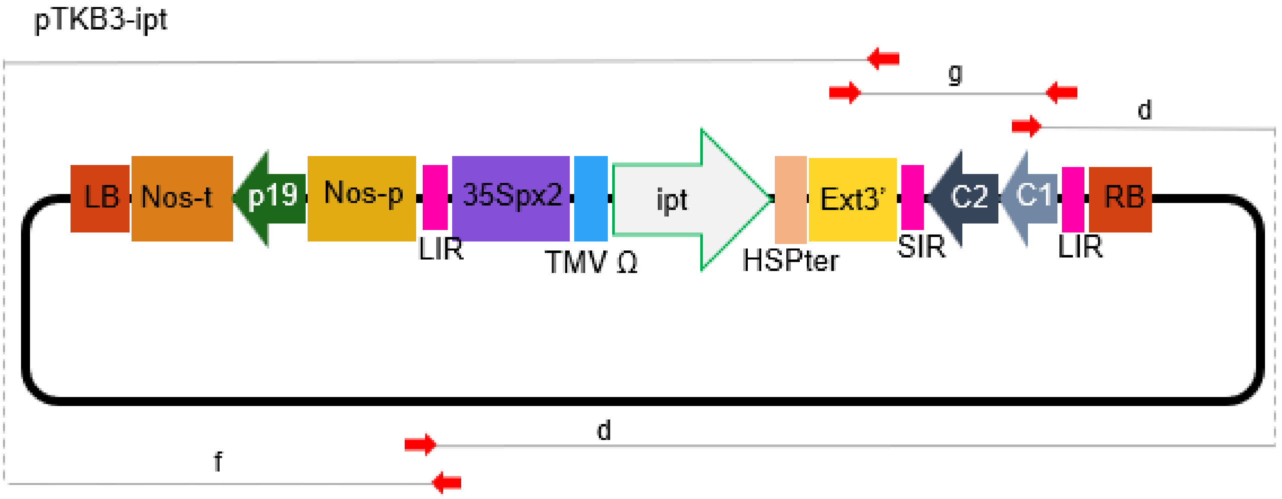

Supplementary Figure S2. Schematic representation of plasmids construction with primers binding sites listed in TableS3. (a) pTKCL-U6(26)-F and izCas9Check3-R; (b) izCas9-2500F and HSPter-R2; (c) izCas9-5500F and RepC1Check1-R; (d) C1-seqF and pTKB3-out-LB-RB-R; (e) pTKB3-out-LB-RB-F and SpaceApaLI-R; (f) pTKB3-out-LB-RB-F and Ext3-R; (g) Ext3-F and RepC1Check1-R

A

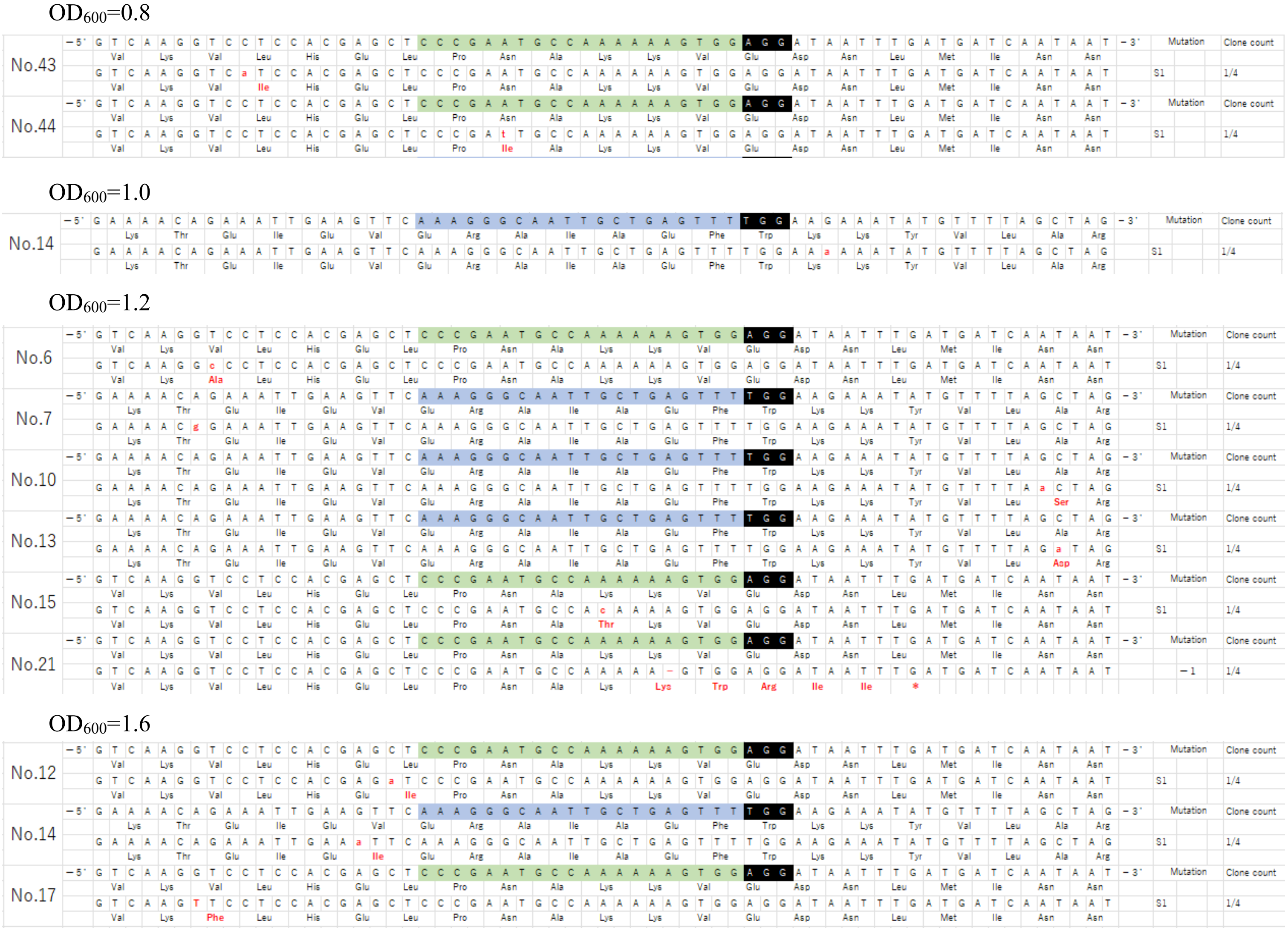
OD600=0.8
No.43
No.44
OD600=1.0
No.14
OD600=1.2
No.6
No.7
No.10
No.13
No.15
No.21
OD600=1.6
No.12
No.14
No.17
Mutation
Clone count

B

Non-post-infection treatment

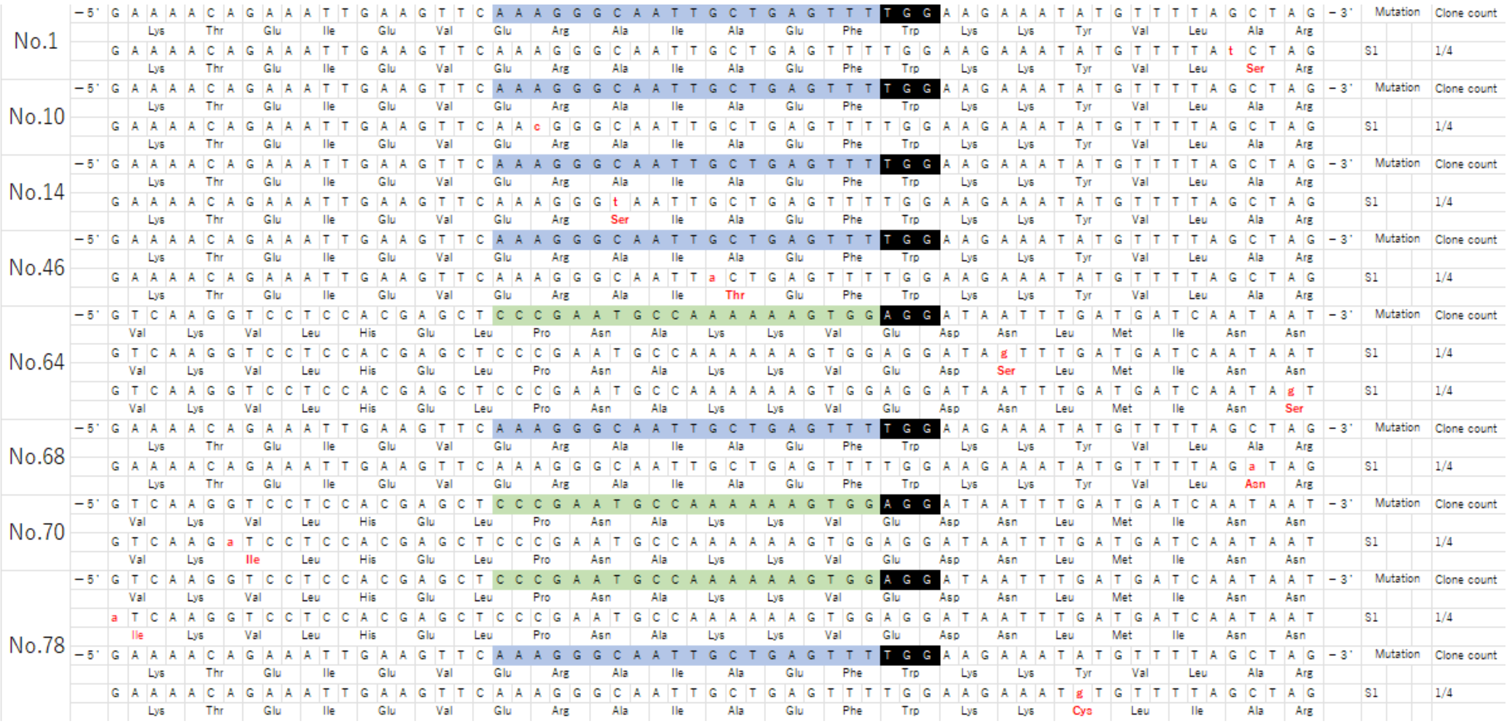

Add liquid fertilizer and 37°C stress (Experiment 1)

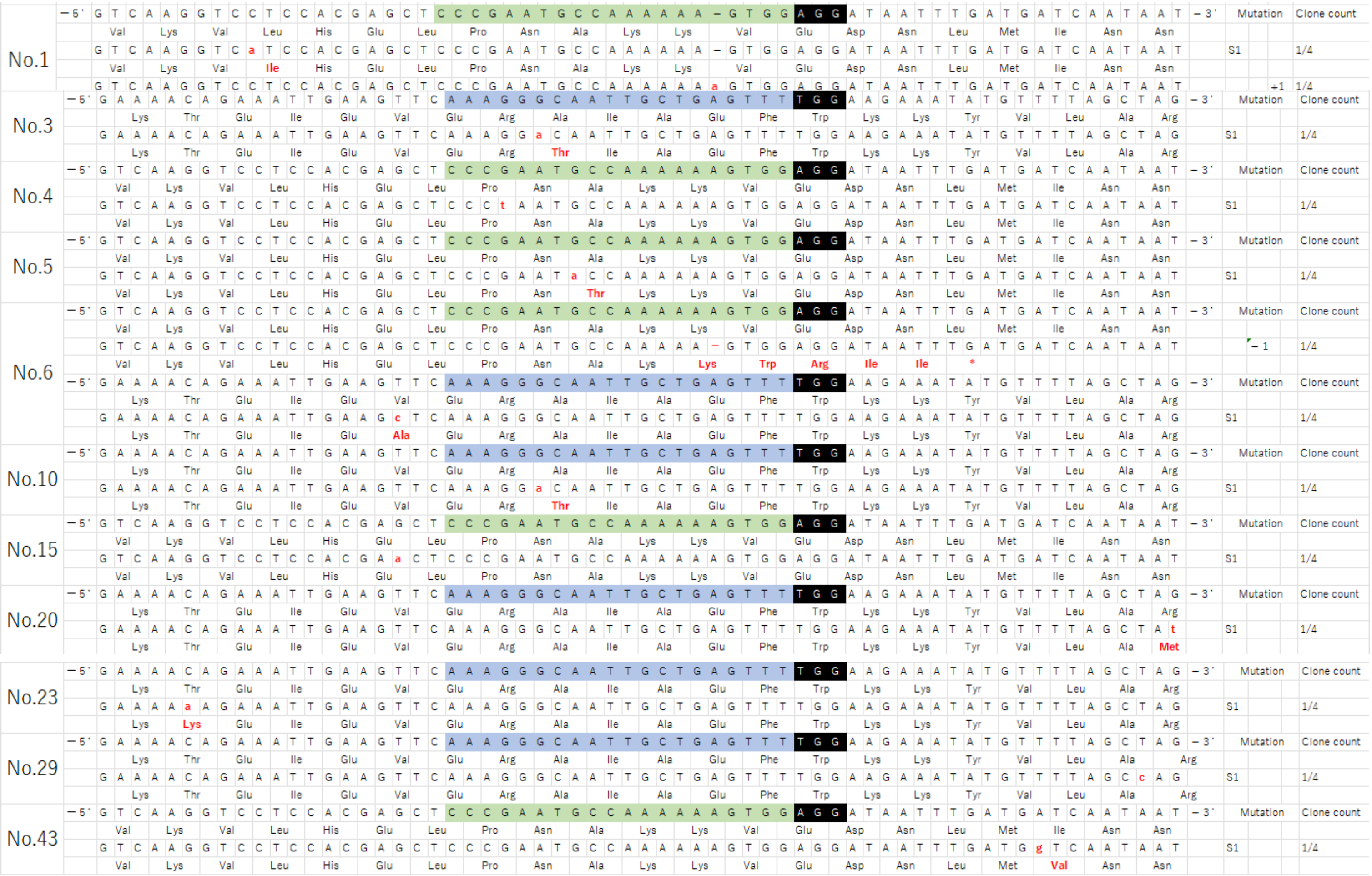

C

Experiment 2

Clone	5'	Sequence	3'	Mutation	Clone count
No.21	−5'	G T C A A G G T C C T C C A C G A G C T C C C G A A T G C C A A A A A A G T G G A G G A T A A T T T G A T G A T C A A T A A T	−3'	Mutation	Clone count
		Val Lys Val Leu His Glu Leu Pro Asn Ala Lys Lys Val Glu Asp Asn Leu Met Ile Asn Asn			
		G T C A A G G T C C T C C A C G A G C c C C C G A A T G C C A A A A A A G T G G A G G A T A A T T T G A T G A T C A A T A A T		S1	1/4
		Val Lys Val Leu His Glu Pro Pro Asn Ala Lys Lys Val Glu Asp Asn Leu Met Ile Asn Asn			
No.44	−5'	G T C A A G G T C C T C C A C G A G C T C C C G A A T G C C A A A A A A G T G G A G G A T A A T T T G A T G A T C A A T A A T	−3'	Mutation	Clone count
		Val Lys Val Leu His Glu Leu Pro Asn Ala Lys Lys Val Glu Asp Asn Leu Met Ile Asn Asn			
		G T C A A G G T C C T C C A C G A G C T C C C G A g T G C C A A A A A A G T G G A G G A T A A T T T G A T G A T C A A T A A T		S1	1/4
		Val Lys Val Leu His Glu Leu Pro Ser Ala Lys Lys Val Glu Asp Asn Leu Met Ile Asn Asn			
No.51	−5'	G T C A A G G T C C T C C A C G A G C T C C C G A A T G C C A A A A A A G T G G A G G A T A A T T T G A T G A T C A A T A A T	−3'	Mutation	Clone count
		Val Lys Val Leu His Glu Leu Pro Asn Ala Lys Lys Val Glu Asp Asn Leu Met Ile Asn Asn			
		G T C A A G G T C C T C C A C G A G C T C C C G A A T G C C A A A A A − G T G G A G G A T A A T T T G A T G A T C A A T A A T		−1	1/4
		Val Lys Val Leu His Glu Leu Pro Asn Ala Lys Lys Trp Arg Ile Ile *			
No.63	−5'	G T C A A G G T C C T C C A C G A G C T C C C G A A T G C C A A A A A A G T G G A G G A T A A T T T G A T G A T C A A T A A T	−3'	Mutation	Clone count
		Val Lys Val Leu His Glu Leu Pro Asn Ala Lys Lys Val Glu Asp Asn Leu Met Ile Asn Asn			
		G T C A A G G T C C T C C A C t A G C T C C C G A A T G C C A A A A A A G T G G A G G A T A A T T T G A T G A T C A A T A A T		S1	1/4
		Val Lys Val Leu His *			
No.65	−5'	G T C A A G G T C C T C C A C G A G C T C C C G A A T G C C A A A A A A G T G G A G G A T A A T T T G A T G A T C A A T A A T	−3'	Mutation	Clone count
		Val Lys Val Leu His Glu Leu Pro Asn Ala Lys Lys Val Glu Asp Asn Leu Met Ile Asn Asn			
		G T C A A G G T C C T C C A t G A G C T C C C G A A T G C C A A A A A A G T G G A G G A T A A T T T G A T G A T C A A T A A T		S1	1/4
		Val Lys Val Leu His Glu Leu Pro Asn Ala Lys Lys Val Glu Asp Asn Leu Met Ile Asn Asn			
No.68	−5'	G A A A A C A G A A A T T G A A G T T C A A A G G G C A A T T G C T G A G T T T T G G A A G A A A T A T G T T T T A G C T A G	−3'	Mutation	Clone count
		Lys Thr Glu Ile Glu Val Glu Arg Ala Ile Ala Glu Phe Trp Lys Lys Tyr Val Leu Ala Arg			
		G A A A g C A G A A A T T G A A G T T C A A A G G G C A A T T G C T G A G T T T T G G A A G A A A T A T G T T T T A G C T A G		S1	1/4
		Lys Ala Glu Ile Glu Val Glu Arg Ala Ile Ala Glu Phe Trp Lys Lys Tyr Val Leu Ala Arg			
No.78	−5'	G T C A A G G T C C T C C A C G A G C T C C C G A A T G C C A A A A A A G T G G A G G A T A A T T T G A T G A T C A A T A A T	−3'	Mutation	Clone count
		Val Lys Val Leu His Glu Leu Pro Asn Ala Lys Lys Val Glu Asp Asn Leu Met Ile Asn Asn			
		G T C A A G G T C C T C C A C G A a C T C C C G A A T G C C A A A A A A G T G G A G G A T A A T T T G A T G A T C A A T A A T		S1	1/4
		Val Lys Val Leu His Glu Leu Pro Asn Ala Lys Lys Val Glu Asp Asn Leu Met Ile Asn Asn			

Clone	5'	Sequence	3'	Mutation	Clone count
No.86	−5'	G T C A A G G T C C T C C A C G A G C T C C C G A A T G C C A A A A A A − G T G G A G G A T A A T T T G A T G A T C A A T A A T	−3'	Mutation	Clone count
		Val Lys Val Leu His Glu Leu Pro Asn Ala Lys Lys Val Glu Asp Asn Leu Met Ile Asn Asn			
		G T C A A G G T C C T C C A C G A G C T C C C G A A T G C C A A A A A A a G T G G A G G A T A A T T T G A T G A T C A A T A A T		+1	1/4
		Val Lys Val Leu His Glu Leu Pro Asn Ala Lys Lys Ser Gly Gly *			
	−5'	G A A A A C A G A A A T T G A A G T T C A A A G G G C A A T T G C T G A G T T T T G G A A G A A A T A T G T T T T A G C T A G	−	Mutation	Clone count
		Lys Thr Glu Ile Glu Val Glu Arg Ala Ile Ala Glu Phe Trp Lys Lys Tyr Val Leu Ala Arg			
		G A A A A C A G A A A T T G A A G T T C A A A G G G C A A T T a C T G A G T T T T G G A A G A A A T A T G T T T T A G C T A G		S1	1/4

Experiment 3

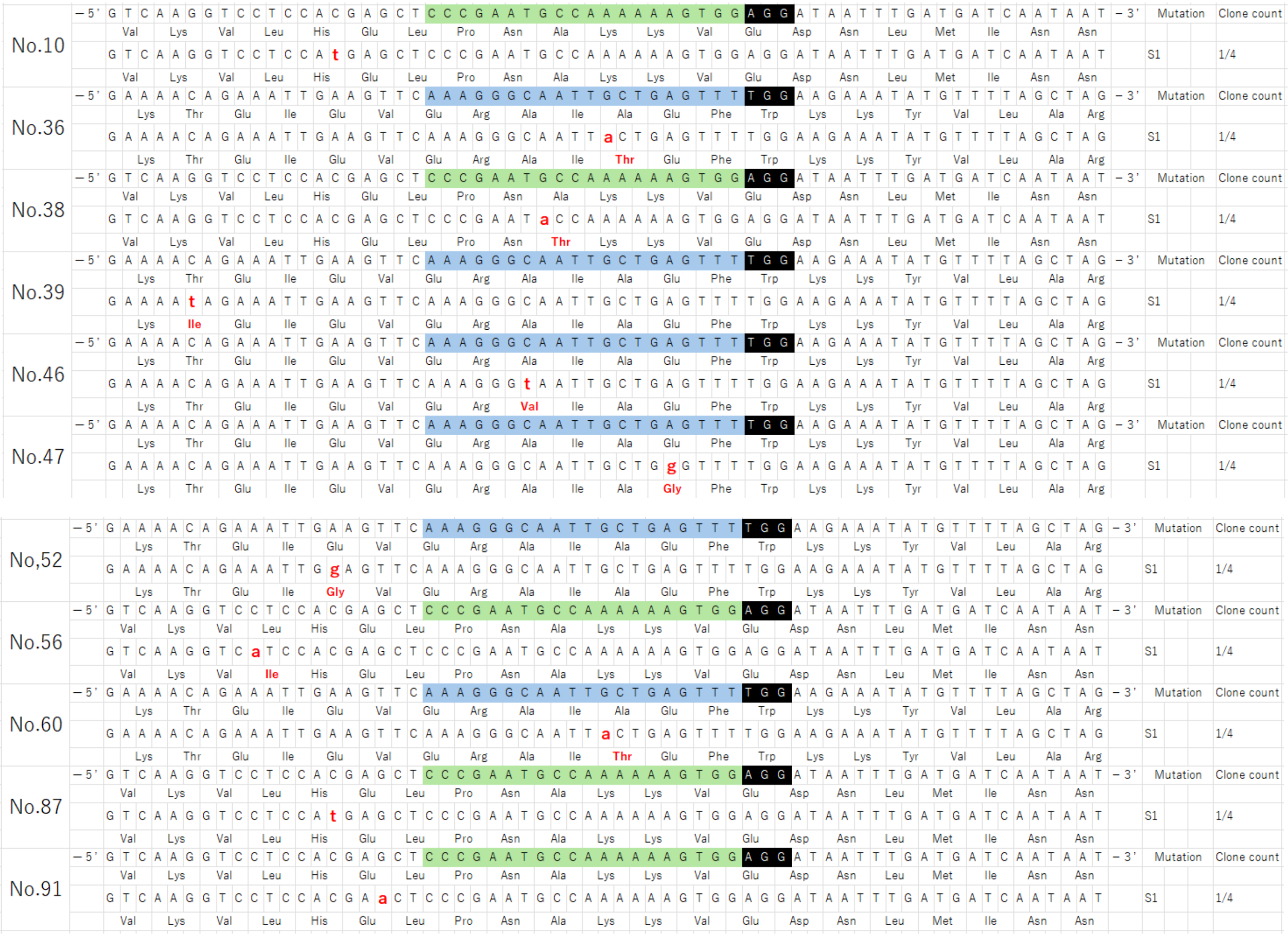

Supplementary Figure S3. Sequencing results for tomato T_0 mutants. Green, Target sequence (Target 1); Blue, Target sequence (Target 2); Black box, PAM sequence; Red, base and amino acid substitutions; Mutation, type of mutation; S, base substitution; -, deletion; +, insertion; Clone count, each individual 4 clones were cloned and used for sequencing analysis. The percentage of mutants found among the analyzed clones is shown.

(A) Infection groups with four Agrobacterium concentrations: details of mutation types are shown in Table 1.

(B) Infection groups used to test post-infection treatments: details of mutation types are shown in Table 2.

(B)-Experiment1 and (C) Infection groups with biological replicates: details of mutation types are shown in Table 3.

ipt-No.30	−5'	GTCAAGGTCCTCCACGAGCTCCCGAATGCCAAAAAAGTGGAGGATAATTTGATGATCAATAAT	−3'	PCR error	Clone count
		Val Lys Val Leu His Glu Leu Pro Asn Ala Lys Lys Val Glu Asp Asn Leu Met Ile Asn Asn			
		GTCAAGGTCCTCCACGAGCTCCCGAAT**A**CCAAAAAAGTGGAGGATAATTTGATGATCAATAAT		S1	1/4

Supplementary Figure S4. Sequence results of shoot with error obtained by infecting only Agrobacterium with pTKB3-ipt. Green, Target sequence (Target 1); Black box, PAM sequence; Red, base substitution; PCR or sequence error, type of error; S, base substitution; Clone count, each individual 4 clones were cloned and used for sequencing analysis. The percentage of errors found among the analyzed clones is shown.

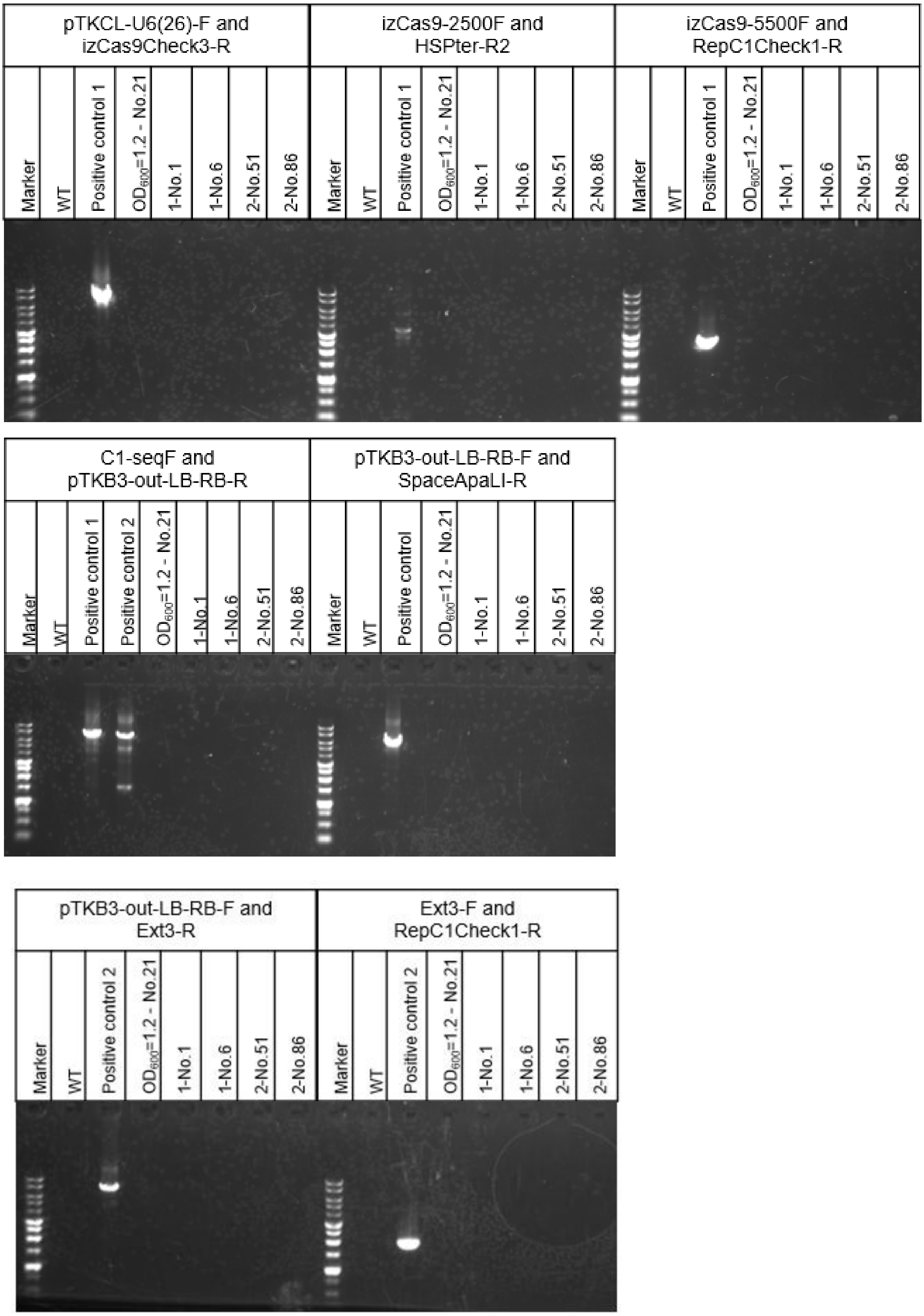

Supplementary Figure S5. Gel electrophoresis of PCR analysis for plasmid insertion in mutants, showing the absence of plasmid integration in mutant T_0 lines. (a) PCR was performed using the primers listed in TableS3: pTKCL-U6(26)-F and izCas9Check3-R; izCas9-2500F and HSPter-R2; izCas9-5500F and RepC1Check1-R; C1-seqF and pTKB3-out-LB-RB-R; pTKB3-out-LB-RB-F and SpaceApaLI-R; pTKB3-out-LB-RB-F and Ext3-R; Ext3-F and RepC1Check1-R. Positive control 1, pTKizCLS-SlGAD3; Positive control 2, pTKB3-ipt. (b) the full uncropped Gel image of PCR analysis electrophoresis

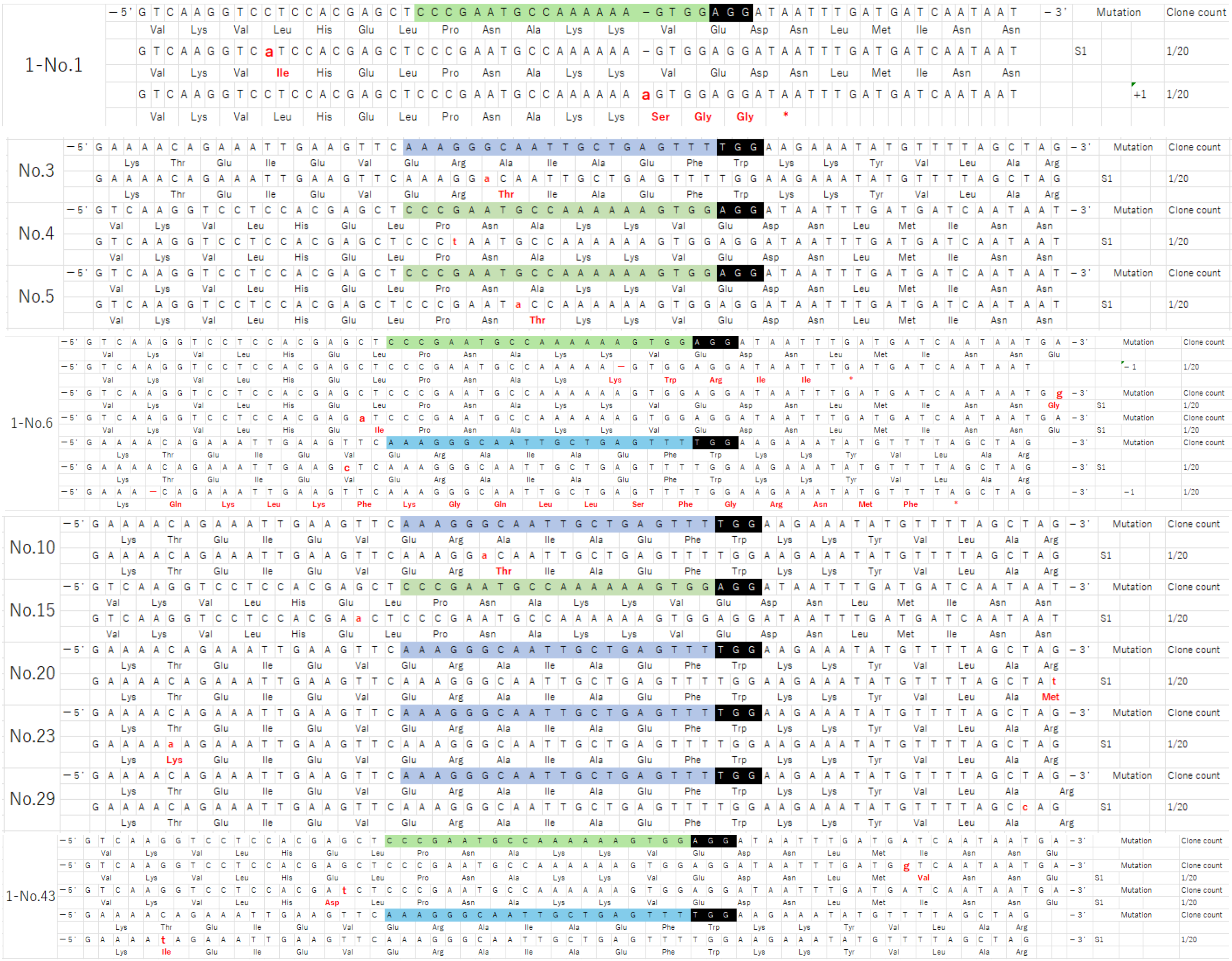

Supplementary Figure S6. Mutation types of T_0 mutants with 20 clones sequenced. Sequencing results of 11mutant lines from experimental group 1 with post-infection treatment of tomato are shown. Green, Target sequence (Target 1); Blue, Target sequence (Target 2); Black box, PAM sequence; Red, base and amino acid substitutions; Mutation, type of mutation; S, base substitution; -, deletion; +, insertion; Clone count, each individual 20 clones were cloned and used for sequencing analysis. The percentage of mutants found among the analyzed clones is shown.

.

Table S1. Primers used for plasmid construction.

Name of primer	DNA sequence (5'-to-3')
**pTKC-LSGAD3**	
pTKCL-U6-F	CCGTCGATCCACTAGCTTTTTTTCTTCTTCTTCGTTC
U6-26-SlGAD3-1-R	CCACTTTTTTGGCATTCGGGCAATCACTACTTCGACTCTAGCTG
SlGAD3 guideRNA F	GCCCGAATGCCAAAAAAGTGGGTTTTAGAGCTAGAAATAGCAAG
U6-26-SlGAD3-2-R	AAACTCAGCAATTGCCCTTTCAATCACTACTTCGACTCTAGCTG
SlGAD3-2 guideRNA-F	AAAGGGCAATTGCTGAGTTTGTTTTAGAGCTAGAAATAGCAAG
pTKCL-U6end-R	ATCACTCCCGACTAGTTTCGCGCAGATTTGCATCCAAAAC
**pTKizCLS-SlGAD3**	
pTKCL-U6-F	CCGTCGATCCACTAGCTTTTTTTCTTCTTCTTCGTTC
pTKCL-U6end-R	ATCACTCCCGACTAGTTTCGCGCAGATTTGCATCCAAAAC

Table S2. Primers for amplification of genomic DNA fragment

Name of primer	DNA sequence (5'-to3')
SlGAD3-multiF	CCCGAACCCTAGCAGATCGTCTTG
SlGAD3-check2R	CTTATTCCTTCAAAACAACCATTAATCC

Table S3. Primers for confirmation of vector persistence

Name of primer	DNA sequence (5’-to3’)
pTKCL-U6(26)-F	CCGTCGATCCACTAGCTTTTTTTCTTCTTCTTCGTTC
izCas9Check3-R	CATGCTGAGAACCTTACGC
izCas9-2500F	ATGACCTGTTGAAAATCATCAAGG
HSPter-R2	TTTAATATTAGAAAAAGCCAC
izCas9-5500F	CTGGTTTTCTACAATTACAGGTGC
RepC1Check1-R	TAGCAAGAACTTCAGACTCCAATC
C1-seqF	ACTTGGGGTAGGTAAGGAAAAC
pTKB3-out-LB-RB-R	GCGGTTTTTCGCTTCTTGGT
pTKB3-out-LB-RB-F	AATCGGACGTTTGACCGGAA
SpaceApaLI-R	AGGTCACTAGCTGTGCACTTC
Ext3-R	TTTTCTCATTGTCTCTCGAAAATG
Ext3-F	TATAAAGAACTGCGCTCTCTG
